# Mastocytose xanthélasmoide: entité rare de mastocytose cutanée

**DOI:** 10.11604/pamj.2017.28.104.13629

**Published:** 2017-10-04

**Authors:** Hind Ramid, Fouzia Hali

**Affiliations:** 1 Service de Dermatologie et de Vénérologie du CHU Ibn Rochd, Casablanca, Maroc

**Keywords:** Mastocytose xanthélasmoide, mastocytose cutanée, signe de Darrier, Xanthelasmoid mastocytosis, cutaneous mastocytosis, Darrier’s sign

## Abstract

Mastocytosis is a rare disease characterized by the abnormal accumulation of mast cells in the skin and possibly in other organs. It can occur in a variety of forms; xanthelasmoid mastocytosis(XM) is a very rare form classified as papulo-nodular. Clinically, it appears as buff-yellow soft papules or nodules of variable size. Triggerings are those of classic mastocytosis. Darier’s sign is often absent. Histology shows dense infiltrate of mast cells in the deep dermis. This clinical form can be singled out because of the persistence of lesions beyond puberty without the additional risk of systemic involvement. We here report the case of a 18-month old female infant, with no previous medical history. Maculopapular, intensely pruritic, ovalaires, brownish lesions with a buff-yellow centre, with elastic consistency and different sizes first occurred at the age of 8 months. The initial interview of parents revealed that the infant had had episode of flush especially caused by warmth. Darrier’s sign was negative. Skin biopsy showed dermal infiltrate of mast cells, allowing to retain the diagnosis of xanthelasmoid mastocytosis. Complementary examinations as well as trypsin dosage were normal. Treatment was based on the exclusion of drugs and of factors participating in mast cells degranulation and antihistamines.

## Image en médecine

La mastocytose est une maladie rare caractérisée par l’accumulation anormale de mastocytes dans la peau et éventuellement dans d’autres organes. Les formes cliniques sont multiples dont la mastocytose xanthélasmoide (MX) qui est une forme très rare classée parmi les formes papulo-nodulaires. Elle se présente cliniquement par l’apparition de papules ou nodules de couleur jaune chamois, de consistance molle, et de taille variable. Les facteurs déclenchants sont ceux d’une mastocytose classique. Le signe de Darier est souvent absent. L’histologie met en évidence un infiltrat dense en mastocytes au niveau du derme profond. Cette forme clinique se singularise par la persistance des lésions au-delàs de la puberté sans sur- risque d’atteinte systémique. Nous rapportons le cas d’un nourrisson de sexe féminin âgée de 18 mois, sans antécédents, dont l’histoire remonte à l’âge de 8 mois par l’apparition de lésions maculo-papuleuses très prurigineuses, ovalaires, brunâtres à centre jaune-chamois, de consistance élastique et de tailles différentes, l’interrogatoire avait objectivé la survenue d’épisode de flush surtout à la chaleur. Le signe de Darrier était négatif. La biopsie cutanée montrait un infiltrat dermique de mastocytes, permettant ainsi de retenir le diagnostic de mastocytose cutanée dans sa forme Xanthélasmoide. Les examens complémentaires ainsi que le dosage de la trypsine étaient normales. Le traitement était basé sur l’éviction de médicaments et facteurs de dégranulation mastocytaires et les antihistaminiques de type anti-H1.

**Figure 1:**
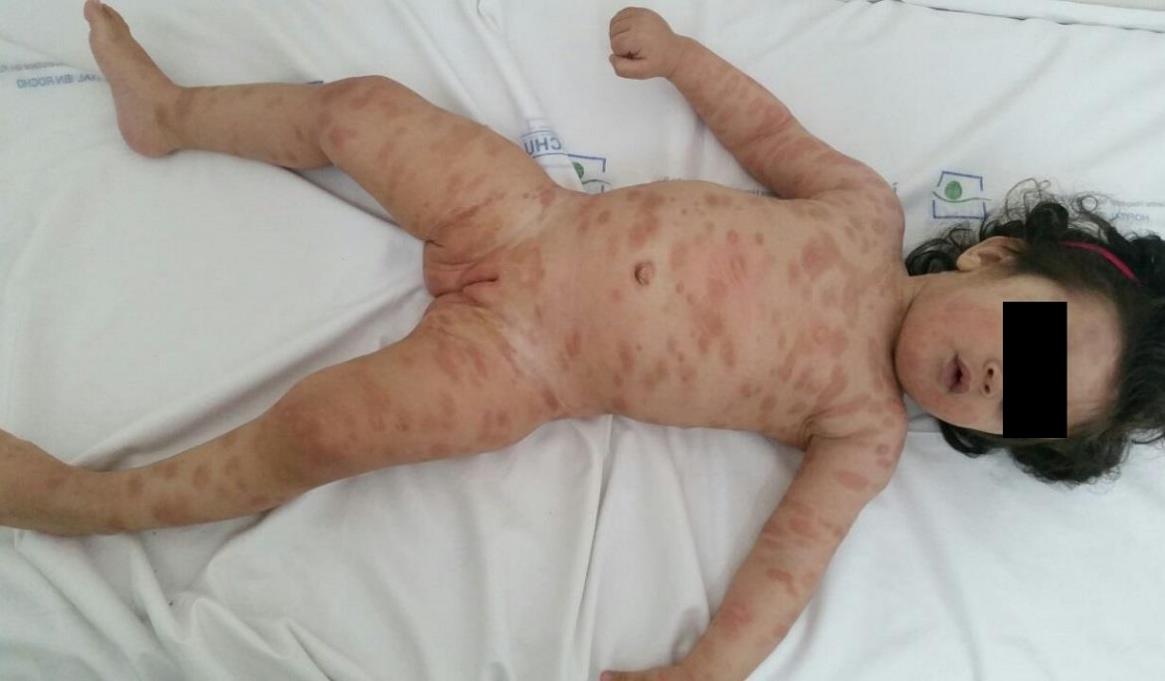
Lésions maculo-papuleuses hyperpigmentées à centre jaune chamois

